# Single-nucleus RNA-seq reveals no increase in T cells in Alzheimer’s disease prefrontal cortex or hippocampus

**DOI:** 10.3389/fncel.2025.1681881

**Published:** 2025-12-15

**Authors:** Jake D. Oxendine, Daniel W. Sirkis, Caroline Jonson, Jennifer S. Yokoyama

**Affiliations:** 1 Edward and Pearl Fein Memory and Aging Center, Department of Neurology, Weill Institute for Neurosciences, University of California, San Francisco, San Francisco, CA, United States; 2 Pharmaceutical Sciences and Pharmacogenomics Graduate Program, University of California, San Francisco, San Francisco, CA, United States; 3 Center for Alzheimer’s and Related Dementias, National Institutes of Health, Bethesda, MD, United States; 4 DataTecnica LLC, Washington, DC, United States; 5 Department of Radiology and Biomedical Imaging, University of California, San Francisco, San Francisco, CA, United States; 6 Global Brain Health Institute, University of California, San Francisco, San Francisco, CA, United States

**Keywords:** CD8 T cells, Alzheimer’s disease, single-nucleus RNA sequencing, ROSMAP, prefrontal cortex

## Abstract

**Background:**

Alzheimer’s disease (AD) has long been associated with hallmark protein aggregates, yet increasing evidence suggests immune involvement may contribute to its progression. Prior studies have found increased T cell presence in AD brain tissue, raising the possibility of neuroimmune crosstalk.

**Methods:**

We used single-nucleus RNA sequencing data from the Religious Orders Study and Memory and Aging Project (ROSMAP), the largest available postmortem AD cohort, to investigate T cell dynamics in prefrontal cortex (PFC) and hippocampus.

**Results:**

Contrary to prior findings, we observed no significant increase in T cell frequency in individuals with pathologically confirmed AD in either region. We replicated these findings in dorsolateral PFC (DLPFC) using the Seattle Alzheimer’s Disease Brain Cell Atlas (SEA-AD). Notably, although we confirmed a prior finding of T cell expansion in middle temporal gyrus (MTG), the strength of this association was affected by donor age. Additionally, we detected no change in gene expression in T cells in the brain parenchyma from individuals with AD.

**Impact:**

These results suggest that T cell enrichment in AD may be regionally restricted and not as widespread as previously assumed. Our findings underscore the importance of brain region selection, analytical approach, and dataset composition in interpreting immune cell dynamics in neurodegenerative disease.

## 1 Introduction

Alzheimer’s disease (AD) is the most common neurodegenerative disease worldwide, affecting nearly 55 million people. AD most commonly occurs in adults above the age of 65, manifesting as changes in memory and other cognitive functions as a consequence of neurodegeneration of brain circuitry occurring downstream of amyloid-*β* plaque and tau neurofibrillary tangle formation (Alzheimer’s Association, 2024). Most research on AD has focused on amyloid-β and tau, but more recently, it has been recognized that inflammation is likely a key player in driving or exacerbating disease pathology.

In the immune system, T cells are central to the adaptive immune response, which targets and eliminates specific pathogens. T cells are divided into two main subtypes: CD4 + and CD8 + T cells. CD4 + T cells regulate immune responses and activate other immune cells. CD8 + T cells, also known as cytotoxic T cells, are involved in the direct destruction of cells and could contribute to neuronal cell death in individuals with AD (DeMaio et al., 2022). Previous research has shown that participants with early-onset AD (EOAD), typically defined as AD with symptom onset before the age of 65, have expansion of interferon-responsive T cells in peripheral blood (Sirkis et al., 2024). On the other hand, participants with late-onset AD have clonal expansion of a subset of effector memory CD8 + T cells (Gate et al., 2020), and cerebrospinal fluid (CSF)-resident T cells in these participants have increased interferon-responsive gene expression (Sirkis et al., 2024). These results suggest differences in T cell inflammatory responses across different tissue compartments in individuals with AD, motivating further exploration of the role of T cells in the brain parenchyma.

T cells have been observed since the early 2000s in the brain via immunohistochemical analysis of postmortem tissue harboring AD neuropathology (Togo et al., 2002). Indeed, multiple studies have reported an increased number of CD8 + T cells in AD brain parenchyma relative to control brain tissue (Togo et al., 2002; Merlini et al., 2018; Chen et al., 2023) and in tauopathy mouse models (Laurent et al., 2017; Lee et al., 2021; Chen et al., 2023). Over the last decade, single-cell and single-nucleus RNA sequencing (snRNA-seq) technology has been used to identify new cell types and determine their relative abundance in postmortem brain tissue (Lee et al., 2021; Mathys et al., 2023; Sun et al., 2023; Yamakawa and Rexach, 2024). With these methods, T cells have been found in increased abundance in both mouse models (Lee et al., 2021; Chen et al., 2023) and human participants with AD (Yamakawa and Rexach, 2024). However, to our knowledge, the question of brain T cell enrichment has not yet been addressed using the largest postmortem AD dataset, the Religious Orders Study/Memory and Aging Project (ROSMAP) (Bennett et al., 2018). The ROSMAP study features 427 participants with postmortem samples containing immune cells from prefrontal cortex (PFC) and has already been utilized to identify cell type abundance and expression differences associated with AD (Mathys et al., 2019, 2023; Sun et al., 2023). Here, we use ROSMAP brain immune cell snRNA-seq data to show that there is no detectable increase in the abundance of parenchymal T cells in PFC or hippocampus of participants with pathologically diagnosed AD and no detectable increase in interferon-responsive gene expression in T cells in the brain parenchyma. In addition, using the Seattle Alzheimer’s Disease Brain Cell Atlas (SEA-AD) (Gabitto et al., 2024), we detect no expansion of T cells in dorsolateral prefrontal cortex (DLPFC) in AD. Importantly, although we could detect the previously reported expansion of T cells in middle temporal gyrus (MTG) using the SEA-AD dataset (Yamakawa and Rexach, 2024), the inclusion of relevant biological covariates could impact the strength of this association. Taken together, our results using the current largest publicly available dataset add important nuance to the literature that T cells are increased in the brain parenchyma of individuals with AD and suggest that important region-specific differences in T cell infiltration exist in AD.

## 2 Materials and methods

All analysis was done in R version 4.5.0 and using Seurat version 5.3.0.

### 2.1 ROSMAP dataset

The primary dataset used for this analysis was the “immune_cells.rds” object obtained from Synapse in January 2024 (syn52368905). Additional analyses were conducted using the “Hippocampus.rds” object obtained from Synapse in September 2024 (syn52408592). This data is derived from postmortem brain tissue from PFC and hippocampus and was processed through the MIT preprocessing workflow, which can be found in Mathys et al. (2023). Samples in this study were classified by pathological diagnosis of AD. *Ethical approval:* All participants enrolled without known dementia and agreed to detailed clinical evaluation and brain donation at death. All studies were approved by an Institutional Review Board of Rush University Medical Center. Each participant signed informed and repository consents and all ROSMAP participants signed the Anatomical Gift Act.

### 2.2 ROSMAP PFC cell type identification

Original cluster labels were provided in the immune_cells object. *T cell assignment*: Upon examination of the previously annotated “T cells,” we determined that this label contained not only T cells but also natural killer (NK) cells, monocytes, and B cells. Therefore, the cells originally labeled as T cells were subclustered (dims 1:30 and resolution = 0.3). One subcluster was removed since most cells in that cluster came from one donor. The remaining subclusters were relabeled using marker genes (*CD3E*, *CD3F*, and *CD3G* for T cells; *CD8A* and *CD8B* for CD8 + T cells; *CD4* for CD4 + T cells; and *NCAM1* for NK Cells). In order to replicate the findings from Sun et al. (2023), the microglia were subclustered with the parameters specified in the original publication (dims = 1:30 and resolution = 0.5). However, because we analyzed the PFC rather than all regions jointly, the resulting clusters were distinct from those generated in Sun et al. Therefore, we used marker genes provided in the original paper (*MYO1E* and *PTPRG*) in conjunction with results from FindMarkers to identify cell types of interest within this dataset.

### 2.3 ROSMAP hippocampus cell type identification

Original cluster labels were provided in the “Hippocampus.rds.” To replicate the immune_cells object, we filtered the whole region object for the cell types present in the original immune_cells object (“T cells,” “CAMs,” and “Mic_”). Next, T cells were identified by subclustering the cells labeled “T cells” with dims = 1:20 and resolution = 1.2. We used the parameters from Yamakawa and Rexach (2024) since the number of T cells was similar to the lymphocytes in that analysis.

### 2.4 SEA-AD dataset

The “Microglia-PVM – MTG” and “Microglia-PVM – DLPFC” datasets from the SEA-AD: Seattle Alzheimer’s Disease Brain Cell Atlas were downloaded in August 2024 from the CellxGene Discover portal^1^.

### 2.5 SEA-AD cell type identification

In order to replicate the findings from Yamakawa and Rexach (2024), the following clustering workflow was used via Seurat NormalizeData and the FindNeighbors/FindClusters functions on the Microglia-PVM object with the following parameters: dims = 1:40, resolution = 1.2. The *CD247* + lymphocytic clusters were subclustered with the following parameters: dims = 1:20, resolution = 1.2, the exact workflow described in the original paper (Yamakawa and Rexach, 2024).

### 2.6 Cell type abundance analysis

To calculate abundance differences, first, the proportion of each cell type was calculated by dividing the number of cells in a given cluster by the total number of immune cells for each donor. Not all individuals had each cell type, so samples with zero counts of a given cell type were eliminated (see Supplementary Table S1 for exact numbers). Chi-squared tests were performed for AD status as well as covariates (sex, age, and postmortem interval [PMI]) to check for potential biases in missing values. A Wilcoxon test was performed to examine the differences in abundance between the AD and control groups. Finally, a multivariate linear regression model was run to compare T cell proportions between AD and controls, adjusting for age, sex, and PMI.

### 2.7 Differential expression and GO analysis

Differential expression analysis was performed to identify potential differences in gene expression between individuals with AD and controls. First, new cell type labels were generated for each cell that combined the cell type label and the disease status of the donor of origin (e.g., “T_cell_AD”). Next, the FindMarkers function using the default settings was used to find differentially expressed genes between cases and controls in that subcluster. To account for correlation within participants, pseudobulk analysis was also performed by first aggregating counts per patient using the AggregateExpression function, then by using the FindMarkers function between conditions with the test parameter of DESeq2 (Love et al., 2014). Gene Ontology enrichment analysis was performed by using AnnotationDbi mapIDs function to map genes to a Bioconductor annotation package that provides genome-wide annotation data for *Homo sapiens*,^2^ (Pagès, 2025). Then the enrichGO function from clusterProfiler was used to perform the enrichment analysis (Xu et al., 2024).

### 2.8 Minimum detectable effect calculation for null results


$$\mathrm{MDE}=(z_{1-\frac{\alpha }{2}}+z_{1-\beta })*\mathrm{SE}(\hat{\beta })$$

To corroborate null findings and account for potential type II error, we calculated the minimum detectable effect (MDE) for AD on the proportion of our target cell type, utilizing a two-sided t distribution, a target power (*β*) of 80% and an unadjusted alpha (*α*) of 0.05. The standard error of the AD term ( $\hat{\beta }$ ) was extracted from the multivariate linear regression model. Then, to provide context, the MDE was divided by the standard deviation of the proportion of our target cell type to show the scale of the MDE in relation to the measured outcome.

## 3 Results

### 3.1 Recapitulation of previously described microglial subtype expansion

**Figure 1:**
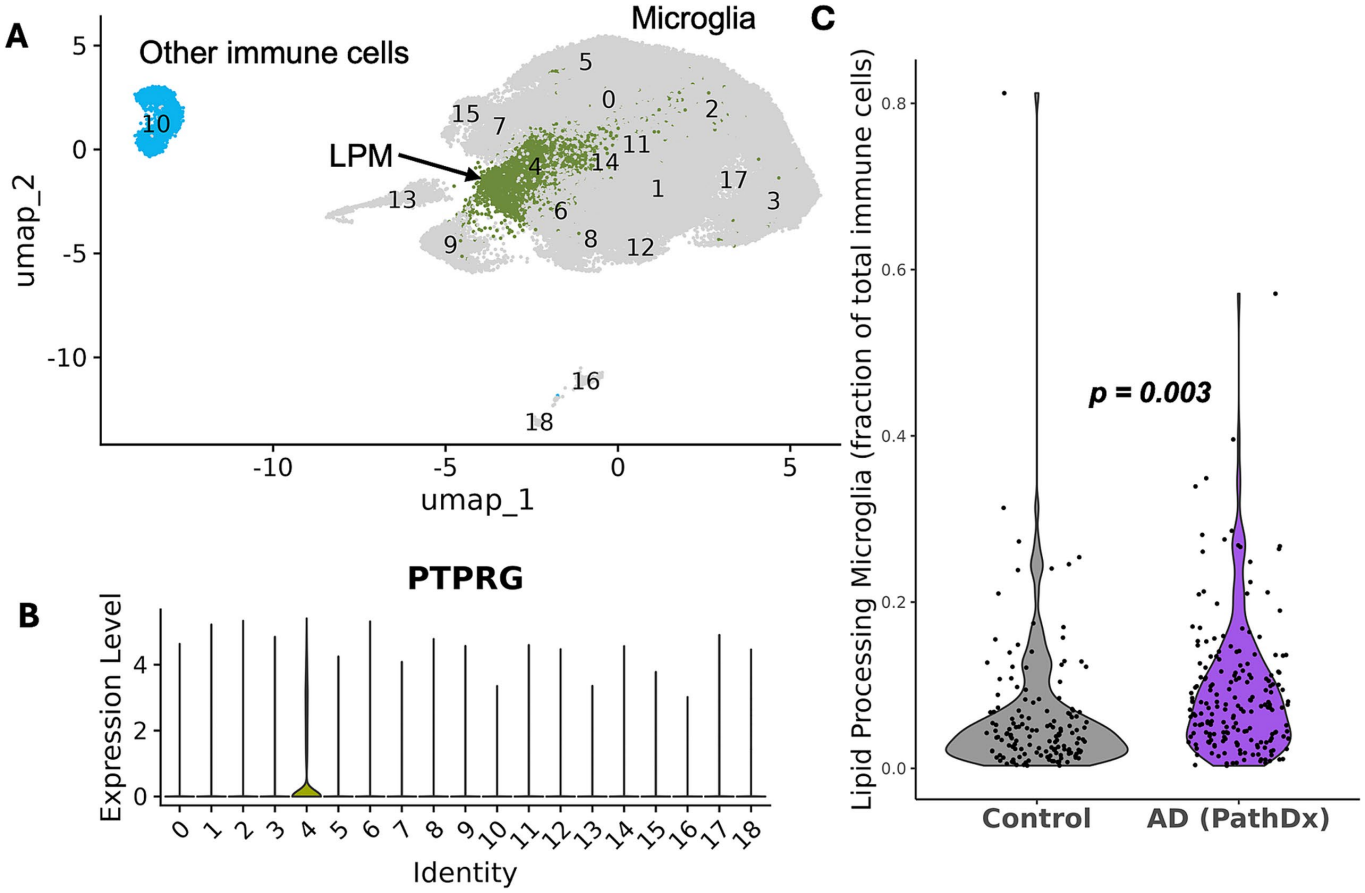
Recapitulation of previously described microglial subtype expansion in AD. **(A)** Uniform manifold approximation and projection (UMAP) plot of ∼161,000 immune cells from PFC from AD cases and pathologically normal controls. Clusters used in analysis (4, LPM [lipid-processing microglia] and 10, cells originally labeled as T cells) are highlighted. Major cell types are labeled within the plot. **(B)** Violin plot of *PTPRG* gene expression by cluster. *PTPRG* is a marker gene of LPM, a subcluster previously found enriched in AD cases by Sun et al., 2023. **(C)** Violin plot showing the fraction of LPM out of total immune cells. LPM are enriched in AD (*p* = 0.003, covariate-adjusted linear regression on 339 participants [21% missing LPM]).

ROSMAP participants with AD have previously been reported to exhibit an expansion of lipid-processing microglia (LPM) defined by marker genes *MYO1E* and *PTPRG* (Sun et al., 2023). We successfully identified this cluster in our version of the dataset (Figures 1A,B) and confirmed its expansion in AD using a Wilcoxon Test (*p* = 5.17 e-07) and through covariate-adjusted linear regression (*p* = 0.0033) (Figure 1C; Table 1). This replication of a previously identified differentially abundant cell type confirmed the validity of our method for assessing T cell abundance.

**Table 1:** Multivariate model results from ROSMAP dataset.

ROSMAP model results								
Lipid processing microglia			PFC CD8 + T cells			Hippocampus CD8+ T cells		
Variable	Estimate	*p*	Variable	Estimate	*p*	Variable	Estimate	*p*
Intercept	0.026	0.35	Intercept	0.014	0.52	Intercept	0.008	0.32
PMI	0.001	0.24	PMI	0.001	0.07	PMI	0.001	0.28
Sex (Male)	0.0005	0.95	Sex (Male)	0.008	0.31	Sex (Male)	−0.0009	0.78
AD (PathDx)	**0.027**	0.0032**	AD (PathDx)	0.001	0.84	AD (PathDx)	0.0002	0.96
Age (75–80)	0.044	0.14	Age (75–80)	−0.004	0.87	Age (80–85)	−0.002	0.76
Age (80–85)	0.019	0.50	Age (80–85)	−0.0021	0.93	Age (85–90)	0.002	0.75
Age (85–90)	0.027	0.32	Age (85–90)	0.009	0.68	Age (90+)	0.002	0.72
Age (90+)	0.033	0.22	Age (90+)	−0.003	0.88			

PMI stands for postmortem interval. Brackets in ranges stand for excluding the last value in the range (ex. [75–80] includes 75, 76, 77, 78, and 79). Significant values are bolded.

### 3.2 No expansion of T cells in PFC in AD in the ROSMAP dataset

**Figure 2:**
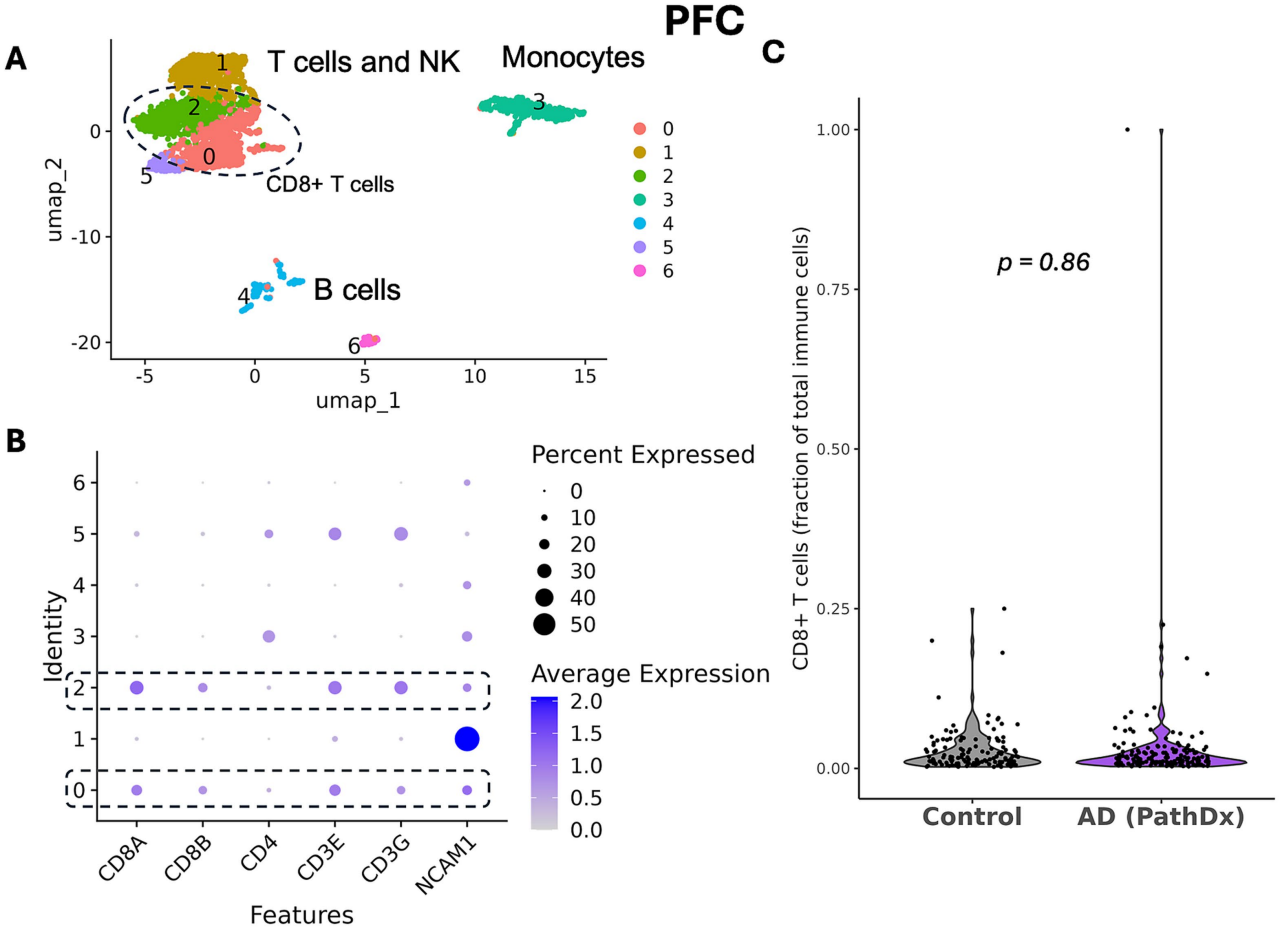
No expansion of CD8 + T cells in PFC in AD in the ROSMAP dataset. **(A)** UMAP plot of 2,534 cells from the T_cells cluster from PFC from AD cases and pathologically normal controls, colored by subcluster identity. Major cell types are labeled within the plot. Circled subclusters were determined to be CD8 + T cells. **(B)** Expression plot of major T cell genes by cluster to determine cell identity. CD8 + T cell subclusters are highlighted. **(C)** Violin plot showing the fraction of CD8 + T cells out of total immune cells (*p* = 0.86, covariate-adjusted linear regression on 300 participants [30% missing CD8 + T cells]).

Following subclustering, the T cell clusters were identified. CD8 + T cells were determined to be clusters 0 and 2, while CD4 + T cells were found in cluster 5 (Figures 2A,B). We first sought to replicate previous findings of expansion of CD8 + T cells (Chen et al., 2023; Yamakawa and Rexach, 2024). We found no expansion of CD8 + T cells in participants with AD by the Wilcoxon test (*p* = 0.47) or by covariate-adjusted linear regression (*p* = 0.86; Figure 2C; Table 1). The MDE is a 0.02 change in the proportion of CD8 + T cells, which corresponds to 34% of the standard deviation in CD8 + T cell proportion. Importantly, we also confirmed that participant samples lacking CD8 + T cells were not enriched for control or AD status, as determined by a chi-squared test (*p* = 1). Several studies had previously reported an expansion of T cells without regard to T cell type (Togo et al., 2002; Merlini et al., 2018; Lee et al., 2021), so we also considered all T cells in aggregate and, again, found no expansion with either the Wilcoxon test (*p* = 0.97) or with the covariate-adjusted linear regression (*p* = 0.27). The missingness of all T cells was once again confirmed not to be due to AD status (*p* = 0.09). Due to the evidence that *APOE* ε4 carrier status can alter immune signatures (Shvetcov et al., 2025), we checked for the impact of *APOE* genotype on our results and found no effect across all analyses (Supplementary Table S1).

### 3.3 No increase in interferon signaling genes in AD

Previous research has shown increased interferon-responsive gene expression in CSF cells from participants diagnosed with AD (Sirkis et al., 2024) and that, in mouse models, blocking interferon signaling impacted progression (Roy et al., 2022; Chen et al., 2023). When performing differential expression analysis, no genes reached multiple testing thresholds for significance in either single-cell or pseudobulk analyses (Supplementary Figures S1A,B). To determine which, if any, pathways may be altered, we took any nominally significant values (*p* < 0.05) either from only the single-cell analysis or from the intersection of the single-cell and pseudobulk analyses and performed Gene Ontology enrichment analysis. We found that interferon signaling was not impacted in T cells in the brain parenchyma from individuals with AD and that the major pathways affected by AD status were related to ubiquitin-protein ligase binding (Supplementary Figure 1C).

### 3.4 No expansion of CD8+ T cells in AD in SEA-AD DLPFC

**Figure 3:**
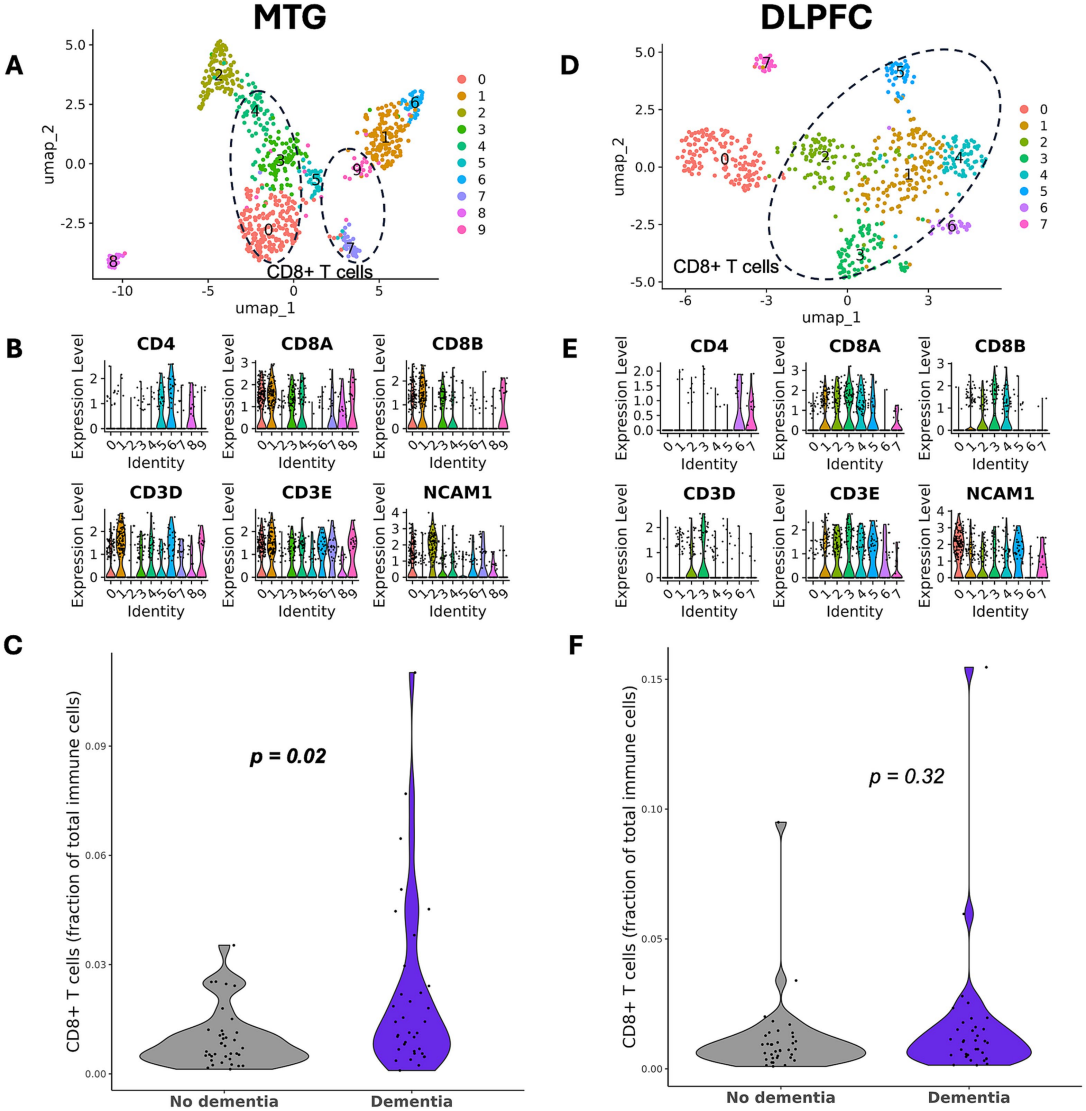
CD8 + T cells are expanded only in MTG of the SEA-AD dataset. **(A)** UMAP plot of 587 CD247 + cells from MTG from AD cases and cognitively normal controls, colored by subcluster identity. Circled subclusters were determined to be CD8 + T cells. **(B)** Expression plot of major T cell genes by subcluster to determine cell identity. Violin plots were used for the SEA-AD dataset due to the smaller number of cells in each subcluster. **(C)** Violin plot showing the fraction of CD8 + T cells out of total immune cells. CD8 + T cells are enriched in MTG in AD (*p* = 0.02, covariate-adjusted linear regression on 72 participants [14% missing CD8 + T cells]). **(D)** UMAP plot of 644 CD247 + cells from DLPFC from AD cases and cognitively normal controls, colored by subcluster identity. Circled subclusters were determined to be CD8 + T cells. **(E)** Expression plot of major T cell genes by subcluster to determine cell identity. **(F)** Violin plot showing the fraction of CD8 + T cells out of total immune cells (*p* = 0.32, covariate-adjusted linear regression on 77 participants [8% missing CD8 + T cells]).

We next sought to replicate our findings in an additional independent dataset. Given that our findings from the ROSMAP dataset were from PFC, the DLPFC dataset from SEA-AD was used. We were able to identify T cell clusters (1, 2, 3, 4, and 5 [CD8+] and 6 [CD4+]) (Figures 3D,E). We successfully replicated our findings from the ROSMAP cohort, showing that there was no significant difference in CD8 + T cell abundance between AD and controls, both by the Wilcoxon test (*p* = 0.20) and by AD status in a multivariate linear regression (*p* = 0.32; Figure 3F; Table 2). The MDE is a 0.014 change in the proportion of CD8 + T cells, which corresponds to 66% of the standard deviation in CD8 + T cell proportion.

**Table 2:** Multivariate model results from SEA-AD dataset.

SEA-AD model results					
MTG			DLPFC		
Variable	Estimate	*p*	Variable	Estimate	*p*
Intercept	**0.021**	0.006**	Intercept	0.012	0.23
PMI (5.9–8.7)	0.001	0.87	PMI (5.9–8.7)	−0.001	0.89
PMI (8.7+)	0.004	0.40	PMI (8.7+)	**0.014**	0.03*
Sex (Male)	−0.003	0.52	Sex (Male)	0.005	0.33
AD (Clinical Dx)	**0.010**	0.02*	AD (Clinical Dx)	0.005	0.32
Age (78–89)	−0.012	0.11	Age (78–89)	−0.003	0.71
Age (90+)	**−0.014**	0.047*	Age (90+)	−0.008	0.32

PMI stands for postmortem interval. Brackets in ranges stand for excluding the last value in range (ex. [75–80] includes 75, 76, 77, 78, and 79). Significant values are bolded.

### 3.5 Replication of CD8+ T cell expansion in SEA-AD MTG

CD8 + T cells have previously been reported to be expanded in MTG using the SEA-AD dataset (Yamakawa and Rexach, 2024). We replicated this finding using the clustering method described in Yamakawa, *et al*. We were able to identify clusters of T cells (0, 3, 4, 7, and 9 [CD8+] and 5 [CD4+]) and filtered out clusters 1 and 6 due to single individuals contributing most of the cells (Figures 3A,B). We found that there was a significant difference with the Wilcoxon test (*p* = 0.010) and in the covariate-adjusted model (*p* = 0.021; Figure 3C; Table 2). However, in this analysis, the age category of 90 + remains significant in the model (*p* = 0.047) (Table 2), a finding which was not previously reported.

### 3.6 No expansion of CD8+ T cells in the hippocampus in AD in the ROSMAP dataset

**Figure 4:**
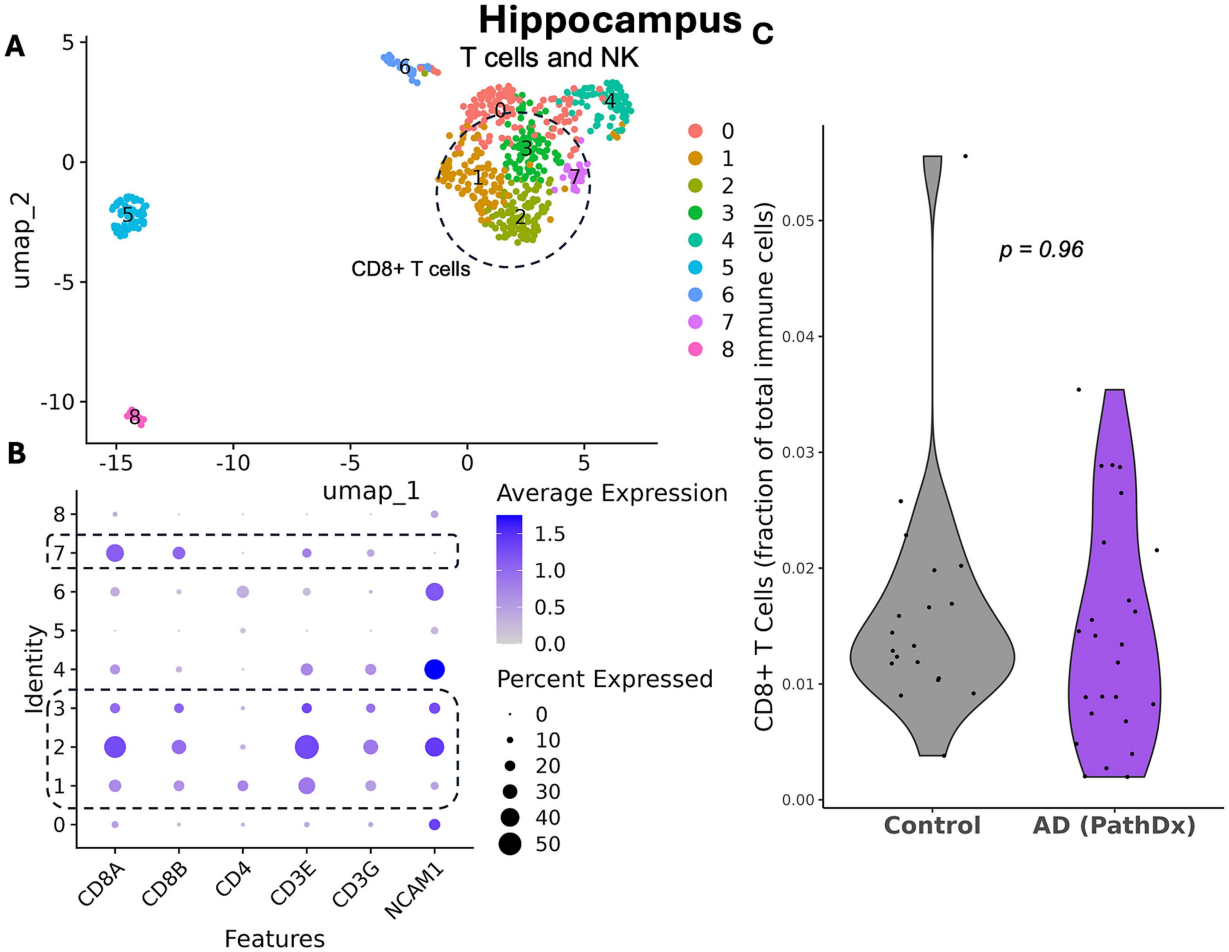
No expansion of CD8 + T cells in hippocampus in AD in the ROSMAP dataset. **(A)** UMAP plot of 608 cells from the T_cells cluster from hippocampus from AD cases and pathologically normal controls, colored by subcluster identity. Major cell types are labeled within the plot. Circled subclusters were determined to be CD8 + T cells. **(B)** Expression plot of major T cell genes by subcluster to determine cell identity. CD8 + T cell subclusters are highlighted. **(C)** Violin plot showing the fraction of CD8 + T cells out of total immune cells (*p* = 0.69, covariate-adjusted linear regression on 44 participants [30% missing CD8 + T cells]).

Finally, we sought to test for expansion of T cells in the hippocampus given that this region is targeted early in the development of AD neuropathology. We were able to identify T cell clusters (1, 2, 3, and 7 [CD8+]; Figures 4A,B). We found that there was no significant difference, either with the Wilcoxon test (*p* = 0.56) or in the covariate-adjusted model (*p* = 0.96; Figure 4C; Table 1). The MDE is a 0.009 change in the proportion of CD8 + T cells, which corresponds to 93% of the standard deviation in CD8 + T cell proportion.

## 4 Discussion

In this study, we found no expansion of CD8 + T cells in the brain parenchyma (specifically in PFC and hippocampus) in samples donated by participants with AD in ROSMAP, the largest postmortem brain dataset for AD. We also replicated this finding in DLPFC using the SEA-AD dataset, another large and widely used dataset. Our results seemingly stand in contrast to the current understanding of the field, where expansion of T cells has been shown through both immunohistochemistry and sc/snRNA-seq in post-mortem brain tissue and animal models (Laurent et al., 2017; Lee et al., 2021; Chen et al., 2023; Yamakawa and Rexach, 2024). With ROSMAP being the largest study of its kind to date, the present negative findings should encourage further investigation of this topic.

Several reasons might account for our results. One possible explanation is the brain region from which the tissue originated. Most studies assessing the question of T cell infiltration in AD have analyzed samples from the hippocampus of both humans and mouse models (Laurent et al., 2017; Lee et al., 2021; Chen et al., 2023; Yamakawa and Rexach, 2024). The hippocampus is associated with memory processing and is one of the first regions affected in AD (Planche et al., 2022). In contrast, PFC, the region from which most of our samples derive, is affected relatively late in the course of disease progression (Planche et al., 2022). It thus remains possible that changes in T cell abundance would be more easily detectable in brain regions affected earlier in the course of disease (such as entorhinal cortex and hippocampus) (Braak and Braak, 1991), which would presumably harbor the most robust pathobiological changes at the end of life. In this regard, it is worth noting that, though we also found no difference in CD8 + T cell abundance in AD in ROSMAP samples derived from the hippocampus, this represents a much smaller dataset than the PFC, and we were unable to replicate the LPM finding from Sun et al. (2023) in the hippocampus (data not shown), suggesting we might be underpowered to detect changes in the hippocampal dataset.

Another potential explanation for our findings relates to the difference in sample size between datasets. While the number of T cells observed per patient is similar to immunohistochemistry studies (Togo et al., 2002; Merlini et al., 2018), the ROSMAP dataset has 427 donors while SEA-AD has 84, much larger than the 8–20 samples typical for immunochemistry studies. The larger datasets may be less subject to outliers and have greater statistical power. A potential downside of these large datasets is the variability introduced by snRNA-seq and the rarity of brain immune cells. Due to sample multiplexing, which limits the number of cells/nuclei per donor and the relative scarcity of non-microglial immune cells in brain tissue, there may be more variability in the number of T cells (and other non-microglial immune cells) detected by snRNA-seq, which makes robust abundance analysis more challenging. To contextualize the robustness of our findings, we have reported MDE sizes for our null results, and we acknowledge that we cannot rule out smaller effects under these limits. Future experimental methods enriching for all non-microglial brain immune cells (e.g., using fluorescence-activated nucleus sorting), may overcome this limitation, and we hope that our findings will encourage validation with immunohistochemistry in different brain regions by others.

Our study suggests the need for more consistent methods and better vetting in single-cell analysis. The first example of this is the use of broad cell type labels. In the downloaded ROSMAP dataset, the initial cluster of cells labeled “T cells” contained several additional classes of peripheral immune cells. It is important that researchers self-assess the validity of any metadata labels provided in publicly available datasets to ensure the robustness of downstream analyses and interpretations. In addition, current clustering techniques are difficult to replicate because they require exact seeds and starting datasets. This makes the process of replicating and reproducing important findings in the field challenging. While we did replicate the findings from Yamakawa and Rexach (2024), clustering differences may have contributed to our finding that age was a significant covariate. Newly developed automated clustering pipelines [e.g., CHOIR (Sant et al., 2025)] may help to enhance replicability and remove subjective measures from single-cell analyses.

This study adds critical nuance to the field’s understanding of the role of the peripheral immune system in AD. Growing evidence has shown that AD is associated with dysregulation of the peripheral immune system. Previous genetic studies have found overlapping genetic variation associated with AD and peripheral immune diseases like Crohn’s disease and psoriasis (Yokoyama et al., 2016). In addition, higher levels of C-reactive protein, IL-6, and other inflammatory markers in healthy adults have been associated with future development of AD (Bettcher et al., 2021). Moreover, in individuals with the strongest AD risk allele, *APOE* ε4, higher levels of inflammatory markers have been associated with earlier onset of disease (Bettcher et al., 2021). AD has also been associated with altered function of peripheral immune cells, including a shift toward pro-inflammatory neutrophils and away from anti-inflammatory signaling in monocytes (Bettcher et al., 2021). Crucially, these peripheral immune markers are also correlated with CSF levels of the major AD biomarkers, amyloid-*β* and phospho-tau (Bettcher et al., 2021).

Our lab has previously reported that T cells in both the peripheral blood and CSF of participants with AD have higher levels of interferon-responsive gene expression, but the present study suggests that this may not be the case in the brain itself. Our results instead support the possibility that dysregulation of the peripheral immune system itself may contribute to AD through peripheral T cell communication with brain-resident immune cells. Thus, our study supports further exploration of the role of the peripheral immune system and its interactions with brain cells in AD. While it is challenging to detect and analyze the limited number of immune cells in existing brain parenchymal snRNA-seq datasets, it might be advantageous to assess potential points of interaction with the peripheral immune system, such as the blood brain barrier, choroid plexus, and meninges, where immune cells are more abundant and crosstalk is thought to occur (Engelhardt and Sorokin, 2009; Yang et al., 2022; Kearns et al., 2023; Reid et al., 2025). Integrating existing brain snRNA-seq datasets with other AD single-cell datasets could also serve as a tool to explore potential crosstalk between the peripheral immune system and the brain. Future studies will be required to test these possibilities.

## Data Availability

All data used in this study are publicly available or can be accessed from a controlled access repository. snRNA-seq data from the MIT ROSMAP Single-Nucleus Multiomics Study (Mathys et al., 2023) is available under controlled access from the AD Knowledge Portal on Synapse under syn52293417; individual-level ROSMAP data is available under syn52430346 and syn3191087. Additional ROSMAP resources can be requested from the Rush Research Resource Sharing Hub at https://www.radc.rush.edu. snRNA-seq data from SEA-AD consortium is publicly available and can be accessed through the CZ CELLxGENE Discover Portal: https://cellxgene.cziscience.com/collections/1ca90a2d-2943-483d-b678-b809bf464c30. Additional SEA-AD resources can be obtained from the AD Knowledge Portal on Synapse under syn26223298. All code used in this analysis can be found at https://github.com/YOKOYAMA-UCSF/Brain-Resident-T-Cells-in-Alzheimer-s-Disease.

## Footnotes

^1^ https://cellxgene.cziscience.com/collections/1ca90a2d-2943-483d-b678-b809bf464c30

^2^ http://org.Hs.eg.db
