# Supplementary material for: Single-nucleus RNA-seq reveals no increase in T cells in Alzheimer’s disease prefrontal cortex or hippocampus
Source: Front Cell Neurosci. 2025 Dec 15;19:1681881. doi: 10.3389/fncel.2025.1681881 (PMC12745470; doi:10.3389/fncel.2025.1681881)
Supplement: Supplementary file 2 [file Presentation_1.pptx]

## Slide 1
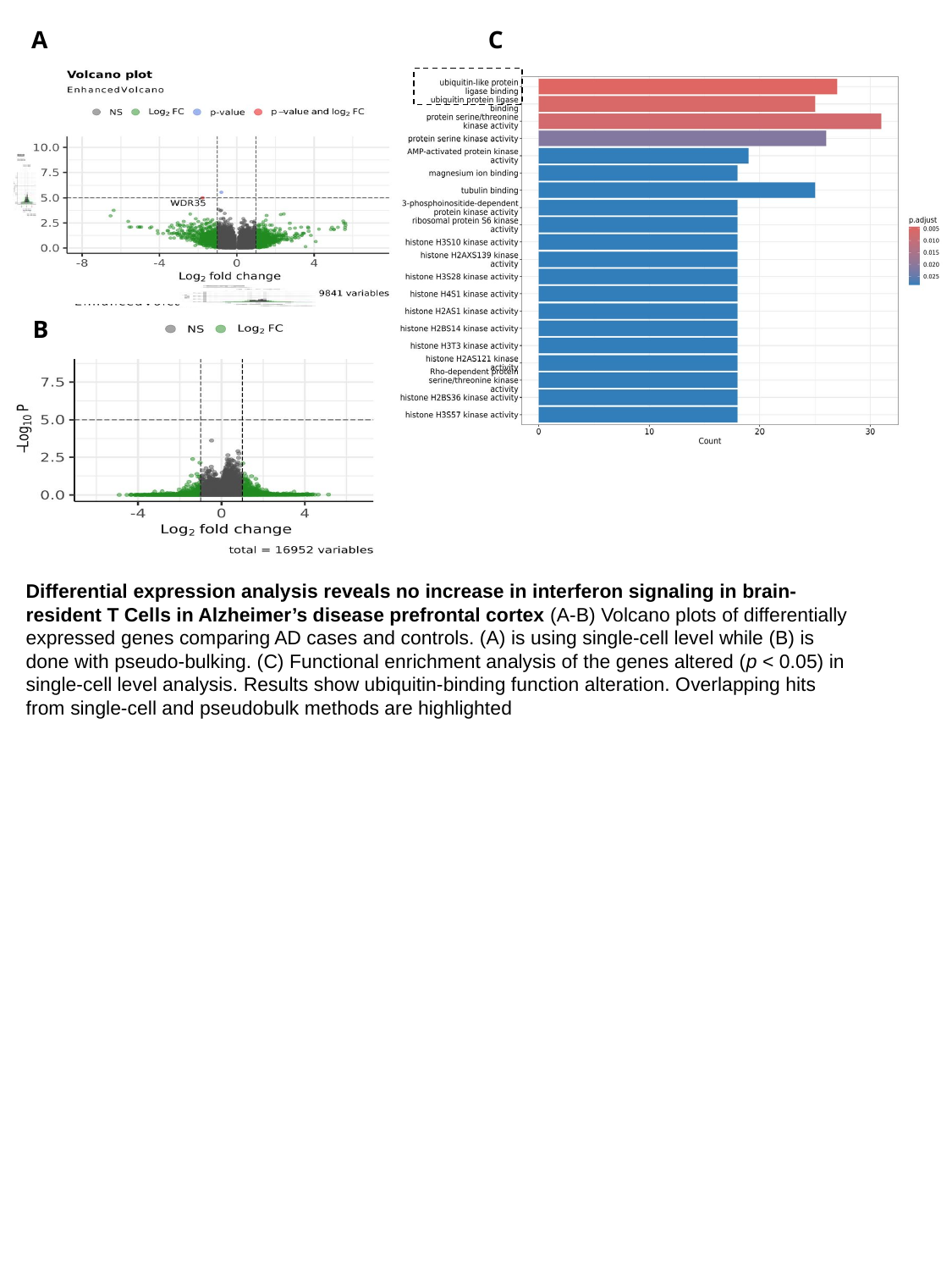

A
C
B
Differential expression analysis reveals no increase in interferon signaling in brain-resident T Cells in Alzheimer’s disease prefrontal cortex (A-B) Volcano plots of differentially expressed genes comparing AD cases and controls. (A) is using single-cell level while (B) is done with pseudo-bulking. (C) Functional enrichment analysis of the genes altered (p < 0.05) in single-cell level analysis. Results show ubiquitin-binding function alteration. Overlapping hits from single-cell and pseudobulk methods are highlighted
